# Study on Flotation Behavior of Fine Flake Graphite Enhanced by Nanobubbles

**DOI:** 10.3390/nano15201542

**Published:** 2025-10-10

**Authors:** Fangyuan Ma, Di Zhang, Dongping Tao

**Affiliations:** 1 School of Mining Engineering, University of Science and Technology Liaoning, Anshan 114051, China; 1209468883@qq.com (F.M.);; 2 School of Resources and Environmental Engineering, Shandong University of Technology, Zibo 255049, China

**Keywords:** nanobubbles, flotation, micro-fine flake graphite

## Abstract

It is difficult to collect fine graphite particles because of the large size and small specific area of traditional flotation bubbles. The contrast experiment between nanobubble flotation and traditional flotation of micro-fine flake graphite in the Hegang area of Heilongjiang Province was carried out in this paper. Under the conditions of feed fineness 78% (−74 μm), pH 10.5, sodium hexametaphosphate 800 g/t, No. 2 oil 350 g/t, pulp concentration 10%, diesel 400 g/t, and pulp cycle time 3 min, the enhanced behavior of nanobubbles on micro-flake graphite flotation was discussed by studying the differences in the flotation rate, selectivity, pulp size, and concentrate size between traditional flotation and nanobubble flotation. The results showed that the nanobubble flotation completed the flotation 20 s earlier than the traditional flotation, and the final concentrate recovery of nanobubble flotation was 1.5 percentage points higher than the traditional flotation. In addition, the average particle size of the slurry from nanobubble flotation is 13 μm larger than that from traditional flotation. In addition, the minimum size of nanobubble flotation is only 2 μm, 11 μm smaller than the minimum size of traditional flotation. Nanobubbles effectively reduce the electrostatic repulsion between fine particles and enhance the hydrophobic attraction, making the hydrophobic aggregates of fine particle graphite more stable. At the same time, the presence of nanobubbles makes the surface hydrophobicity of graphite stronger, effectively promoting the recovery of fine particle graphite.

## 1. Introduction

The reserves of flake graphite in China are relatively rich, but the fine flake graphite resource will become a major resource in the future with the continuous exploitation of the large flake graphite resource [1]. Because fine flake graphite ore contains fine flake graphite less than 38 μm, such as fine flake graphite in the Luobei area of Heilongjiang Province, contains more than 50% of fine flake graphite [2], it is difficult to recover in the flotation process. In recent years, researchers have developed some enhanced flotation techniques for fine flake graphite. Kang et al. showed that compared with conventional flotation, ultrasonic pretreatment technology could increase the grade and recovery of fine flake graphite concentrate by 3.22 percentage points and 9.15 percentage points [3]. Bu et al. reported that the recovery of fine flake graphite recovered by column flotation technology was nearly 10 percentage points higher than that of a mechanical flotation machine [4]. Some researchers have shown that emulsified diesel is more conducive to adsorption on graphite surfaces than conventional diesel, which effectively improves the recovery of fine flake graphite. For example, Sun et al. reported a new oil-in-water kerosene emulsion, which increases the recovery of fine flake graphite by 3 percentage points, and reduces the kerosene consumption by 26–34% [5]; Ma et al. showed that nanobubble flotation could effectively improve the recovery effect of micro-fine flake graphite, increase the recovery by 14.73 percentage points and significantly shorten the number of flotation stages [6]. But no researchers have studied the strengthening behavior of nanobubbles on the flotation of micro-fine flake graphite so far. Therefore, the study of the effect of nanobubbles on the flotation of fine flake graphite is of great significance for the resource utilization of graphite resources.

Nanobubbles have a remarkable enhancement effect on the flotation of micro-fine flake graphite, but there is still a lack of in-depth discussion on the flotation behavior of micro-fine flake by nanobubbles. The micro-fine flake graphite ore from Heilongjiang Province was taken as the research object in this study, and the effect of nanobubbles on the flotation strengthening behavior of micro-fine flake graphite was studied in detail, including flotation rate difference, pulp particle size difference, and concentrate particle size difference, recovery difference, and flotation selectivity, which provided theoretical guidance for the flotation of micro-fine flake graphite.

## 2. Experimental

### 2.1. Materials

#### 2.1.1. Mineral Composition

The test samples were taken from the grinding products of roughage concentrate from a concentrator in the Luobei Area, Heilongjiang Province, and the mineral composition in the samples was obtained by MLA analysis, which is shown in Table 1. As can be seen from Table 1, the fixed carbon content in the sample is 34.93%, and the main gangue mineral content in the muscovite and quartz samples is 16.32% and 22.31%, respectively. The lamellar structure of muscovite is easy to form interlayer structure with flake graphite, which will affect the liberation of graphite [1,6]. It is worth noting that the sample contains 5.23% pyrite, which has good floatability and may easily enter concentrate products in the flotation process, thus reducing concentrate grade.

#### 2.1.2. Particle Size Composition

**Figure 1:**
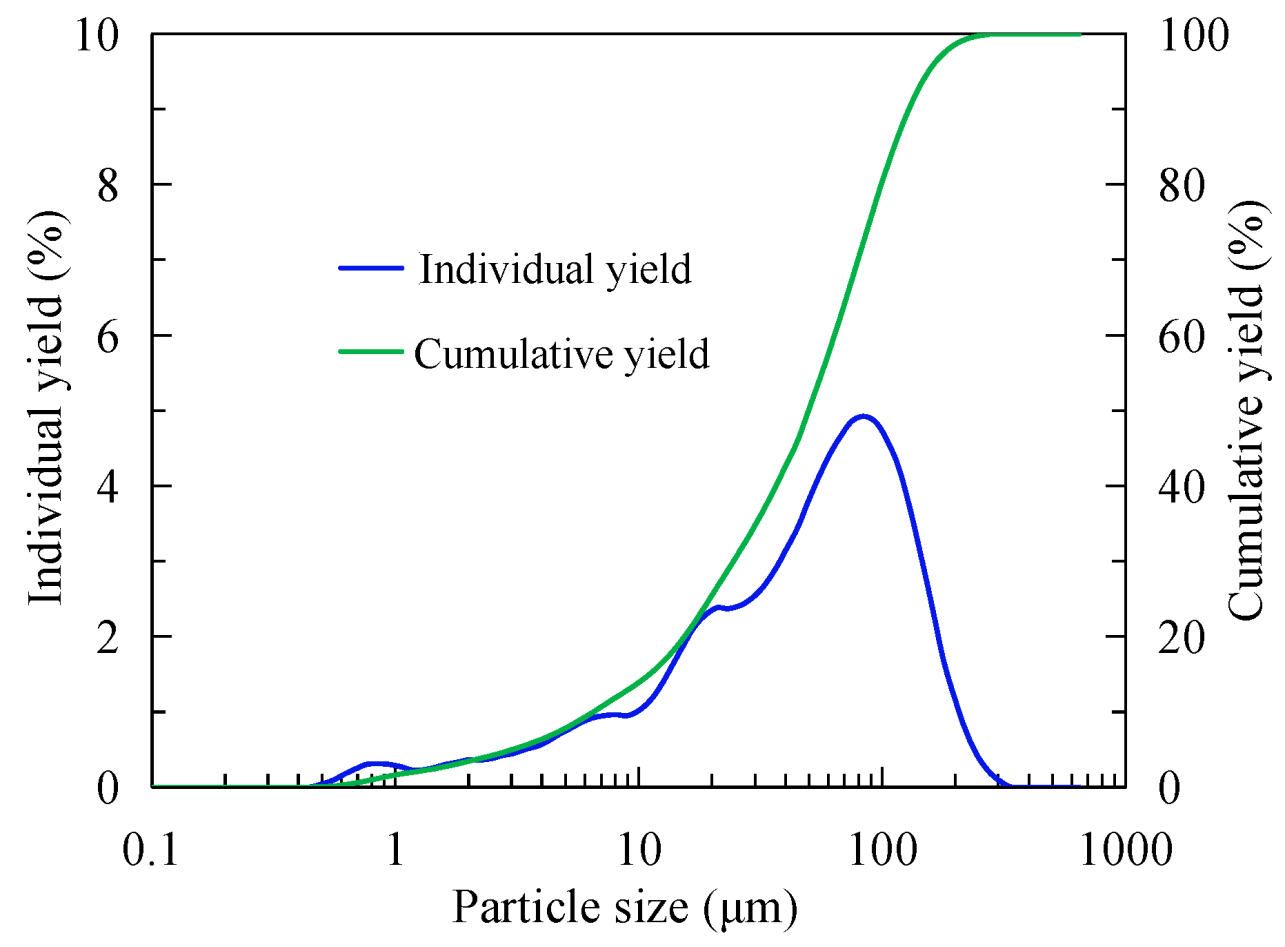
Laser particle size test results of the sample.

The particle size of the sample was analyzed by a laser particle size analyzer (BT-9300S) manufactured by Bettersize Instrument Ltd., Dandong, China, as shown in Figure 1. It can be seen from Figure 1 shows that most samples are concentrated in the range of 50–100 μm. The cumulative yield curve in Figure 1 that fine particles of −20 μm account for about 20% and −74 μm account for 68%. In addition, it also can be seen from the cumulative curve that d50 is about 46 μm and d80 is about 100 μm.

### 2.2. Nanobubble Generation Methods

**Figure 2:**
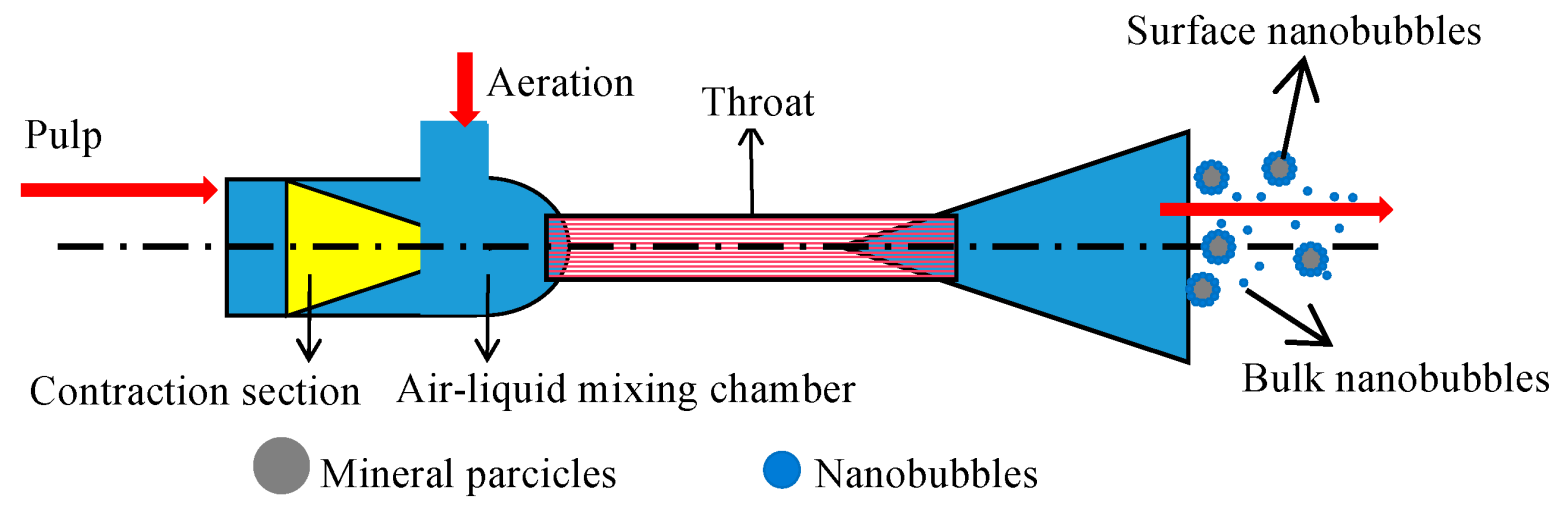
Principle of nanobubbles generated by hydraulic cavitation [11].

Nanobubbles refer to tiny bubbles with a diameter less than 1 μm [7], which can be produced in many ways, such as the solution exchange method, heat exchange method, ultrasonic method, and hydraulic cavitation method [8,9]. Hydraulic cavitation is the simplest, cleanest, and most efficient method to prepare nanobubbles, which is widely used in the flotation of fine particle minerals [10]. In this study, the hydraulic cavitation method is adopted to generate nanobubbles with the help of a bubble generator (Figure 2). When the slurry passes through the pipe of the bubble generator with a throat diameter of 2 mm, the water pressure decreases sharply with the increase in the flow rate, resulting in the air dissolved in water being generated on the particle surface or in the liquid phase in the form of gas nuclei or nanobubbles [11]. At present, the generation of nanobubbles by this method has been confirmed. For example, Oliveira et al. [12] and Zhang et al. [13] successfully prepared nanobubbles by hydraulic cavitation.

### 2.3. Test Equipment

**Figure 3:**
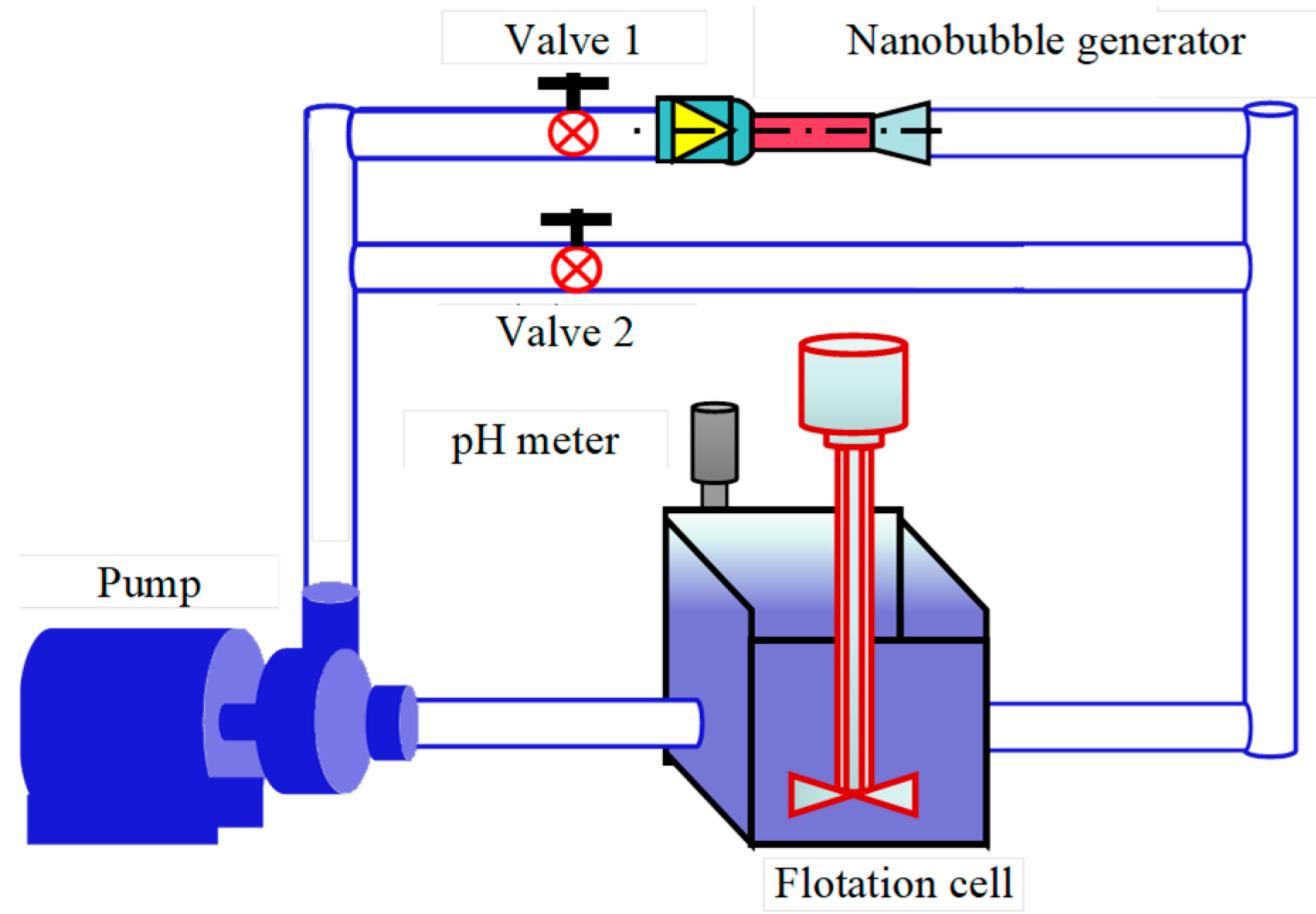
Test device diagram.

The circulating pump and flotation machine (1 L cell) are integrated through the bubble generator and ordinary steel pipe to form the test device (Figure 3). When the slurry passes through the nanobubble generator (valve 1 is opened, valve 2 is closed), nanobubbles will be formed on the surface of graphite based on the principle of hydraulic cavitation. At this time, a nanobubble flotation test can be carried out. On the contrary, when the pulp does not pass through the nanobubble generator (close valve 1, open valve 2), the traditional flotation test can be carried out at this time. By comparing the flotation indexes of the above two conditions, the behavioral differences between nanobubbles and traditional flotation can be studied, which can provide data support for strengthening the flotation behavior of micro-fine flake graphite by nanobubbles. Some scholars have used this device to study the difference between nanobubble flotation and traditional flotation on the flotation behavior of fine particle minerals. For example, Sobhy and Tao have studied the effect of nanobubbles on the flotation of fine particle coal based on a similar device [14], and Sobhy et al. have studied the effect of nanobubbles on the reverse flotation of fine hematite based on this device [15].

### 2.4. Experimental Condition

Based on the device in Figure 3, all tests were conducted under the following conditions: feed fineness of 78% (−74 μm, reference plant), pH 10.5 (quicklime regulation), sodium hexametaphosphate 800 g/t (depressor), No. 2 oil (frother) 350 g/t, pulp concentration 10%, diesel (collector) 400 g/t, and pulp cycle time 3 min. Differences in nanobubble flotation and traditional flotation were investigated, including the difference in flotation rate, difference in flotation selectivity, size difference in flotation pulp, and size difference in flotation concentrate. It should be noted that in addition to regulating pH, quicklime is easy to form Fe(OH)_2_ and Fe(OH)_3_ hydrophilic films or hydrate films of CaSO_4_, CaCO_3_, and CaO on the surface of pyrite [16], which can depress pyrite in samples and is beneficial to the improvement of concentrate grade (Figure 3).

### 2.5. Particle Size Characterization

#### 2.5.1. Pulp Particle Size Measurement

Under the flotation test conditions in Section 2.4, the pulp was reduced to obtain nanobubble flotation pulp samples and traditional flotation pulp samples. 5 mL of the above two types of pulp samples were taken using disposable plastic syringes and placed, respectively, under an optical microscope and in a laser particle size analyzer (BT-9300S) for pulp particle size characterization to evaluate the differences in pulp particle size between nanobubble flotation and the traditional flotation system.

#### 2.5.2. Particle Size Characterization of Flotation Concentrate

Under the flotation test conditions in Section 2.4, nanobubble flotation concentrate products and traditional flotation concentrate products were obtained. Representative samples were obtained through suction filtration, drying, and reduction. Finally, the particle size composition of the above two concentrates was characterized by the screening method, respectively, and then the recovery effects of nanobubble flotation and traditional flotation on fine particle graphite were evaluated. It is worth noting that the hydraulic analysis is adopted because particles smaller than 20 μm cannot be obtained by sieving.

### 2.6. Zeta Potential Measurement

High-purity graphite with 99.99% fixed carbon of −0.043 mm was adopted as the test material. The surface potential of graphite particles was tested with the device in Figure 3 in the presence or absence of nanobubbles. Test conditions: Pulp concentration is 5 mg/L, diesel is 5 mg/L, MIBC concentration is 5 mg/L, and pulp with and without nano-bubbles is obtained after 3 min of pulp circulation. Then, 2 mL of the pulp sample was taken with a disposable syringe for potential testing. Finally, the surface potential of graphite under different pH conditions was obtained.

### 2.7. XPS Characterization

High-purity graphite with 99.99% fixed carbon of −0.043 mm was adopted as the test material. The content changes of oxygen-containing groups on the graphite surface after diesel adsorption in the presence and absence of nanobubbles were studied by using the XPS (Thermo Fisher Inc.) analytical method. Based on the device in Figure 2, pulp samples with and without nanobubbles were obtained under the conditions of a diesel dosage of 5 mg/L, a pH of 10, MIBC of 5 mg/L, and a cavitation time of 3 min. It should be noted that Ma et al.’s (2021) [2] research indicates that the optimal pH value for the flotation of fine flake graphite is around 10 to 11. Therefore, a pH value of 10 is selected for the preparation of XPS test samples [12]. Then, the above samples were, respectively, filtered, washed with distilled water, dried at 40 °C for 20 h, and reduced for XPS analysis. Finally, peak C was subjected to peak separation treatment to obtain the content differences in hydrophobic groups (oxygen-containing groups such as C=O, C-O, COOH) and hydrophilic groups (non-oxygen-containing groups such as C-H, C-C) on the graphite surface.

## 3. Results and Discussion

### 3.1. Flotation Rate

**Figure 4:**
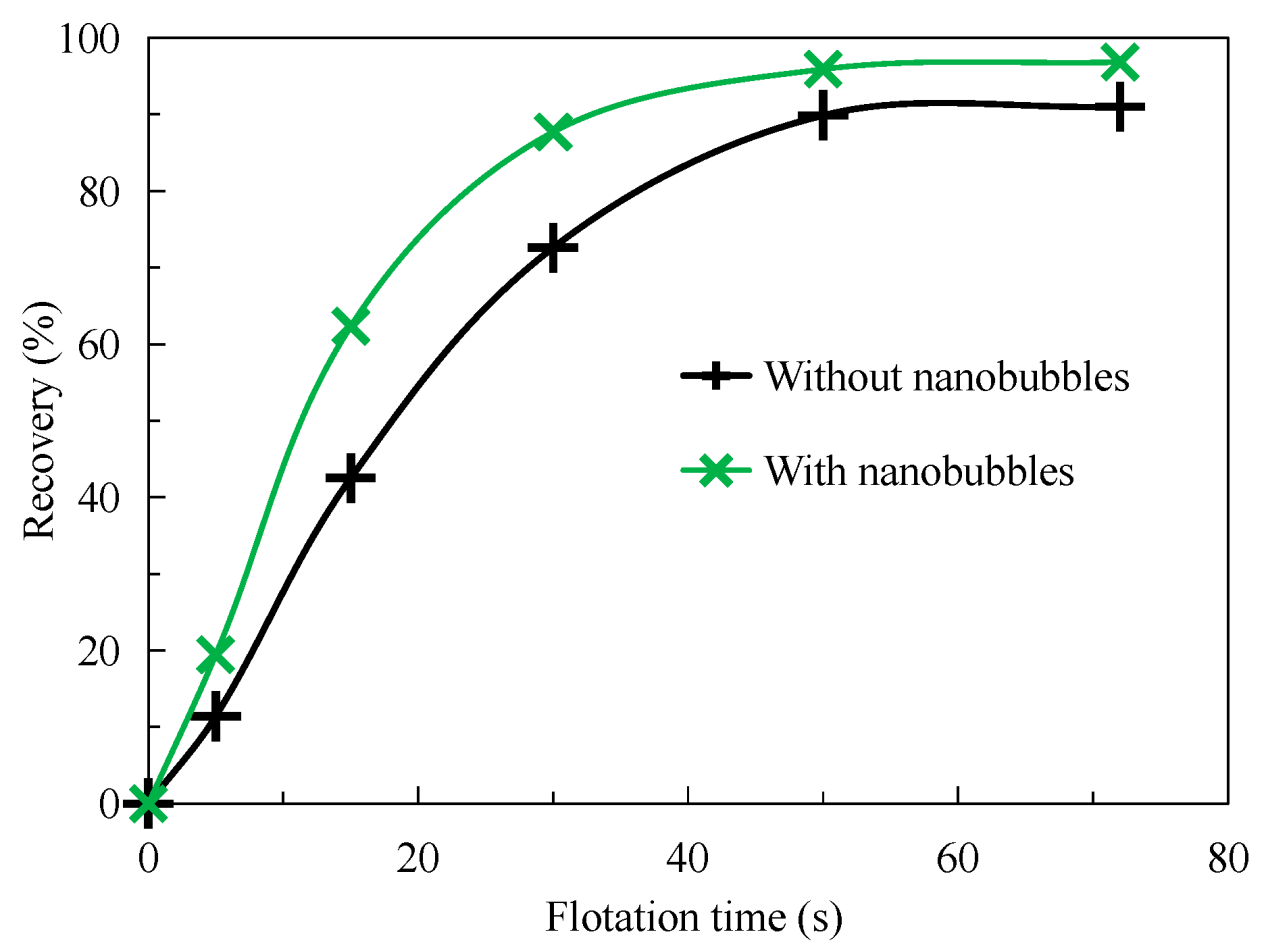
Relation between flotation time and recovery with or without nanobubbles.

Figure 4 shows the results of the flotation dynamics test. As can be seen from Figure 4, the recovery of graphite increases significantly with the increase in flotation time, but the flotation rate of graphite by nanobubbles is significantly higher than that by traditional flotation. For example, when the flotation time is less than 15 s, the nanobubble flotation rate is significantly higher than the traditional flotation. When the flotation time reaches 15 s, the recovery of the nanobubble flotation concentrate reaches 62%, while the recovery of the traditional flotation concentrate is only 43%; that is, the nanobubble flotation recovery is 19 percentage points higher than the traditional flotation concentrate recovery. Greater than 30 s, the recovery of nanobubble flotation concentrate is close to a fixed value of about 89%, while the recovery of traditional flotation concentrate is close to a fixed value of about 88% when the flotation time is about 50 s. Nanobubble flotation is completed 20 s earlier than the traditional flotation operation, which effectively improves the flotation efficiency. When the flotation time reaches 70 s, the recovery of nanobubble flotation concentrate is always about 5 percentage points higher than that of traditional flotation concentrate. Nanobubbles increase not only the kinetics of flotation but also the final efficiency. In fact, compared with traditional flotation, other researchers have confirmed that nanobubbles can improve the flotation rate. For example, Tao et al. found that nanobubble flotation shortened the flotation time from 3 min to 1 min in reverse flotation of ultra-fine hematite [17]. The main reason why the flotation rate of nanobubbles is faster is that the bubble concentration of nanobubbles in the pulp system is higher, which can increase the collision probability between bubbles and particles, effectively mineralizing fine mineral particles and thus accelerating the flotation rate.

The bulk nanobubbles and particle surface nanobubbles are formed after hydraulic cavitation. The increase in flotation rate by bulk nanobubbles can be explained from three aspects. Firstly, the flotation rate depends on the flotation probability, which is the product of the collision probability between particles and bubbles, the probability of particle detachment on the bubble surface, and the probability of particle adhesion on the bubble surface. The flotation probability equation shows that the smaller the bubble size, the higher the mineralization efficiency of mineral particles [10]. Secondly, nanobubbles can exist in the liquid phase for a long time, for several days or even months [18], and have a higher collision probability with mineral particles, which is conducive to the mineralization of mineral particles. Thirdly, another important aspect of nanobubbles in flotation is related to their quantity, density, or concentration in pulp. According to Meyer and Deglond [19], the agglomeration or flotation model of particle collision can be expressed as Formula (1). In flotation, *N*_k_, *N*_i_, and *N*_j_ respectively represent the number density of bubble-particle adhesion aggregates, bubbles, and particles. α represents collision efficiency; beta represents collision frequency, that is, the number of collisions per unit volume and time. The higher the bubble concentration, the higher the collision frequency between particles and bubbles, that is, the faster the flotation rate. Etchepare et al. [20] reported that nanobubble concentration produced by a centrifugal multi-phase pump was as high as 4 × 10 ^9^/mL, and the number of these nanobubbles was much higher than the number concentration of microbubbles under the same conditions; that is, the increase in bubble number density would improve the collision frequency of bubble particles, thus increasing the flotation rate. The surface nanobubbles formed by hydraulic cavitation nucleate out on the hydrophobic particle surface, eliminating the collision process between bubbles and particles, which is another main reason why the flotation rate of nanobubbles is faster than that of traditional flotation [15].

(1) $$\frac{\partial N_{k}}{\partial t}=\alpha \cdot \beta \cdot N_{i}\cdot N_{j}$$

### 3.2. Flotation Selectivity

**Figure 5:**
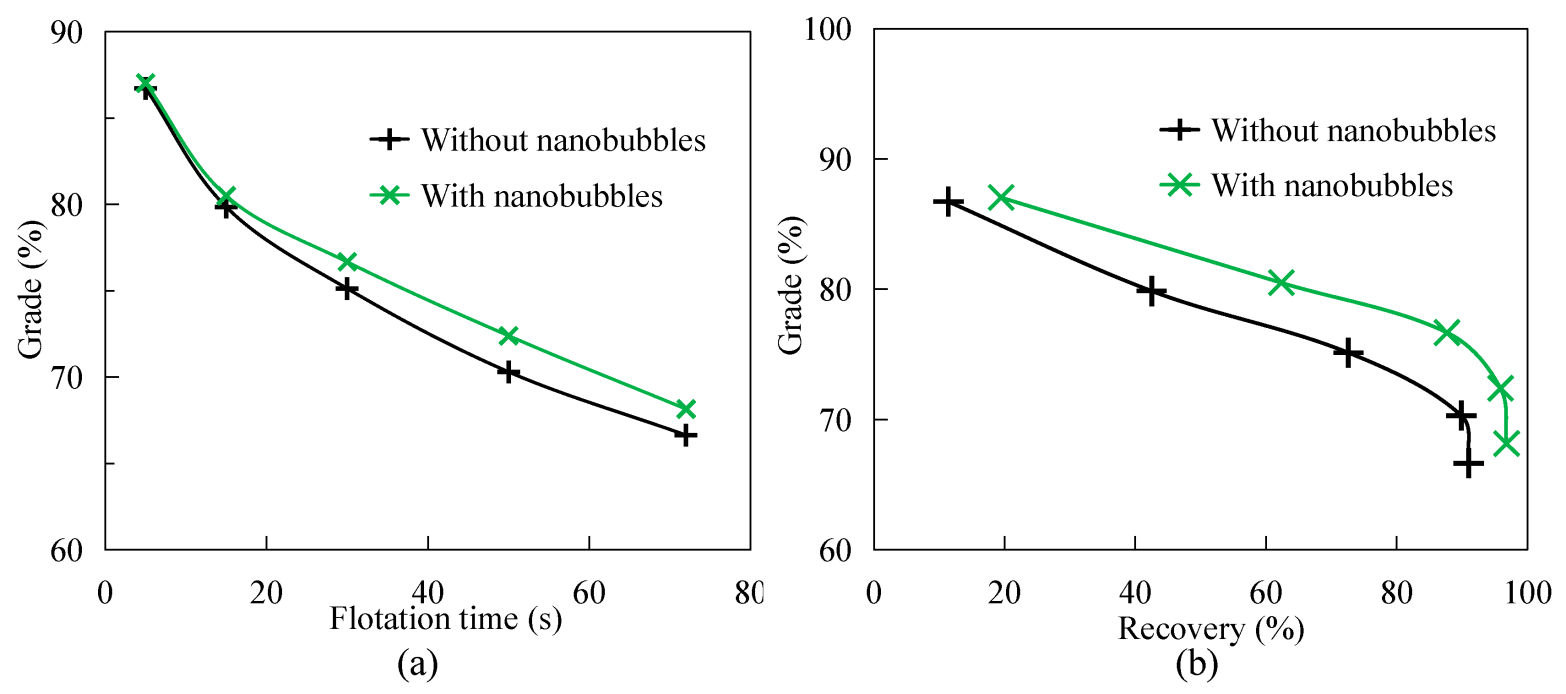
Difference in flotation selectivity between nanobubble flotation and traditional flotation. (**a**) The relationship between grade and flotation time. (**b**) The relationship between grade and recovery rate.

Figure 5 shows the difference in selectivity between nanobubble flotation and conventional flotation. Figure 5a shows that concentrate grade decreases with the extension of flotation time, because the shorter the flotation time, the higher the hydrophobicity of pulp products, and the higher the grade of concentrate products. It can also be seen from Figure 5a that with the increase in flotation time, the grade of nanobubble flotation concentrate is always higher than that of traditional flotation concentrate. For example, when the flotation time is 30 s, the grade of nanobubble flotation concentrate is about 77%, while the grade of traditional flotation concentrate is only 75%; that is, the grade of nanobubble flotation concentrate is 1.5 percentage points higher than that of traditional flotation concentrate. Similarly, when the flotation time is 50 s and 70 s, the grade of nanobubble flotation concentrate is 2 and 1.5 percentage points higher than that of traditional flotation concentrate, respectively. Figure 5b shows that the grade of nanobubble flotation concentrate is always higher than that of traditional flotation concentrate when the recovery of flotation concentrate is the same. For example, when the flotation recovery is about 73%, the traditional flotation concentrate grade is about 70%, while the nanobubble flotation concentrate grade is about 79%; that is, the nanobubble flotation concentrate grade is about 4 percentage points higher than the traditional flotation concentrate grade. In addition, when the flotation recovery is about 42%, the grade of nanobubble flotation concentrate is still 3.5 percentage points higher than that of traditional flotation concentrate. In conclusion, the selectivity of nanobubble flotation is always higher than that of nanobubble flotation in the flotation process, which has a significant strengthening effect on the improvement of flotation concentrate grade.

**Figure 6:**
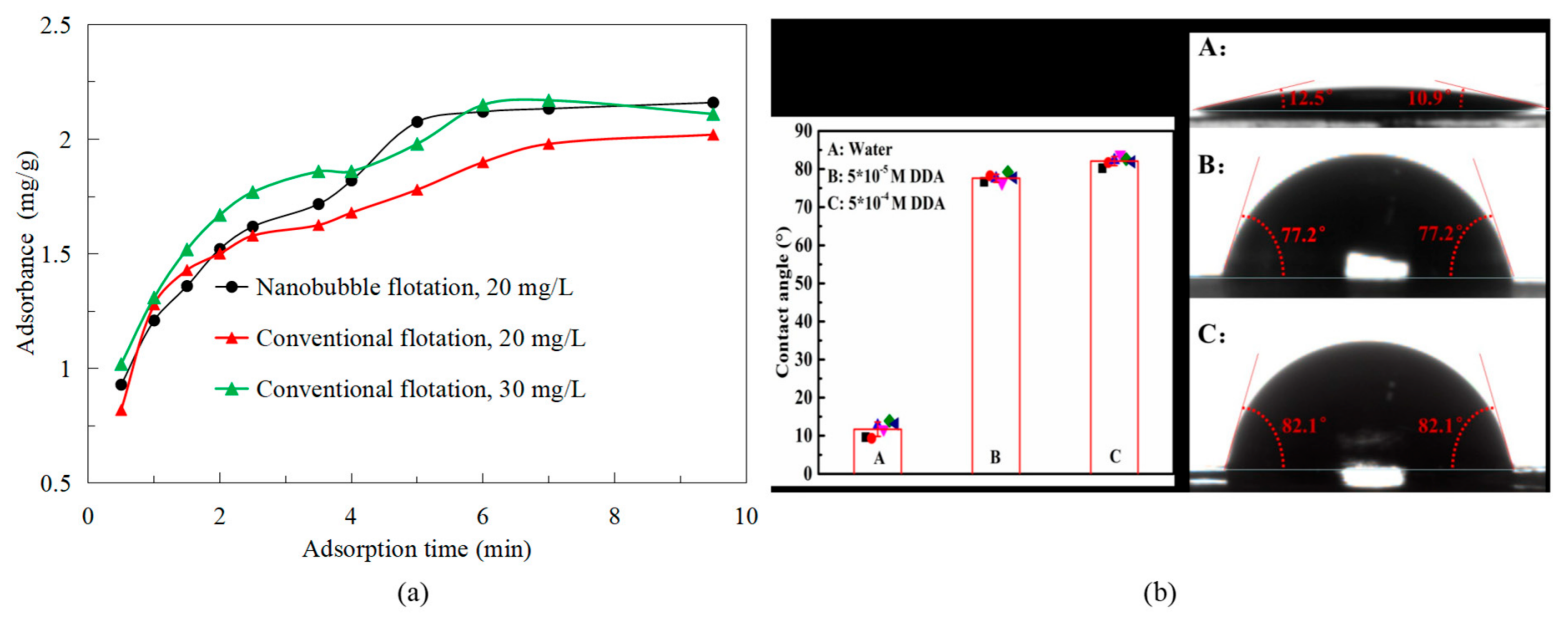
The promoting adsorption curve of the collector on the surface of graphite [21] and the adhesion law of nanobubbles on the surface of muscovite [23].

In fact, the main reason for improving the selectivity of flotation is that nanobubbles enhance the hydrophobicity of mineral surfaces. Ma et al. showed that nanobubbles promoted the adsorption of the collector (diesel) on the graphite surface at low collector concentration, enhanced the adsorption rate of graphite to the collector, and the adsorption capacity was higher than that of the conventional flotation system (Figure 6a), that is, rapidly promoted the surface of graphite to a stronger hydrophobicity in a short time [21]. In addition, Tang et al. [22] found that the contact angle of nanobubble flotation concentrate surface was about 12° higher than that of traditional flotation concentrate by the contact angle test of nanobubble flotation concentrate and traditional flotation concentrate. Then, they used XPS analysis to confirm that the nanobubbles could increase the hydrophobicity of the graphite surface by covering the oxygen-containing groups (hydrophilic groups) [22]. Nanobubbles cover the hydrophilic groups on the mineral surface, increasing the active sites for interaction with the collector and thereby increasing the contact angle of the mineral surface. This further enhances the selectivity of the flotation process.

Of course, nanobubbles promoting collector adsorption have been demonstrated in other mineral flotation. For example, Chen et al. confirmed that under the condition of low concentration of dodecylamine collector, nanobubbles can still promote the adsorption of dodecylamine on micro-fine muscovite surface to obtain a surface with high hydrophobicity [23], thus saving the collector dosage. In addition, the selectivity of nanobubble flotation is also shown in the selective adhesion of nanobubbles to particle surfaces. Zhou et al. confirmed that after hydrophobic treatment with 10^−5^ and 10^−4^ mol/L dodecylamine solution on the muscovite surface, the contact angle increased from 10.9°~12.5° to 77.2°~82.1° (Figure 6b) [24]. On the other hand, the more hydrophobic the mineral surface is, the more likely it is to have nanobubbles. Therefore, the more the surface hydrophobicity of graphite, the more likely the nanobubbles are to adhere to graphite particles, and the flotation selectivity is better.

**Figure 7:**
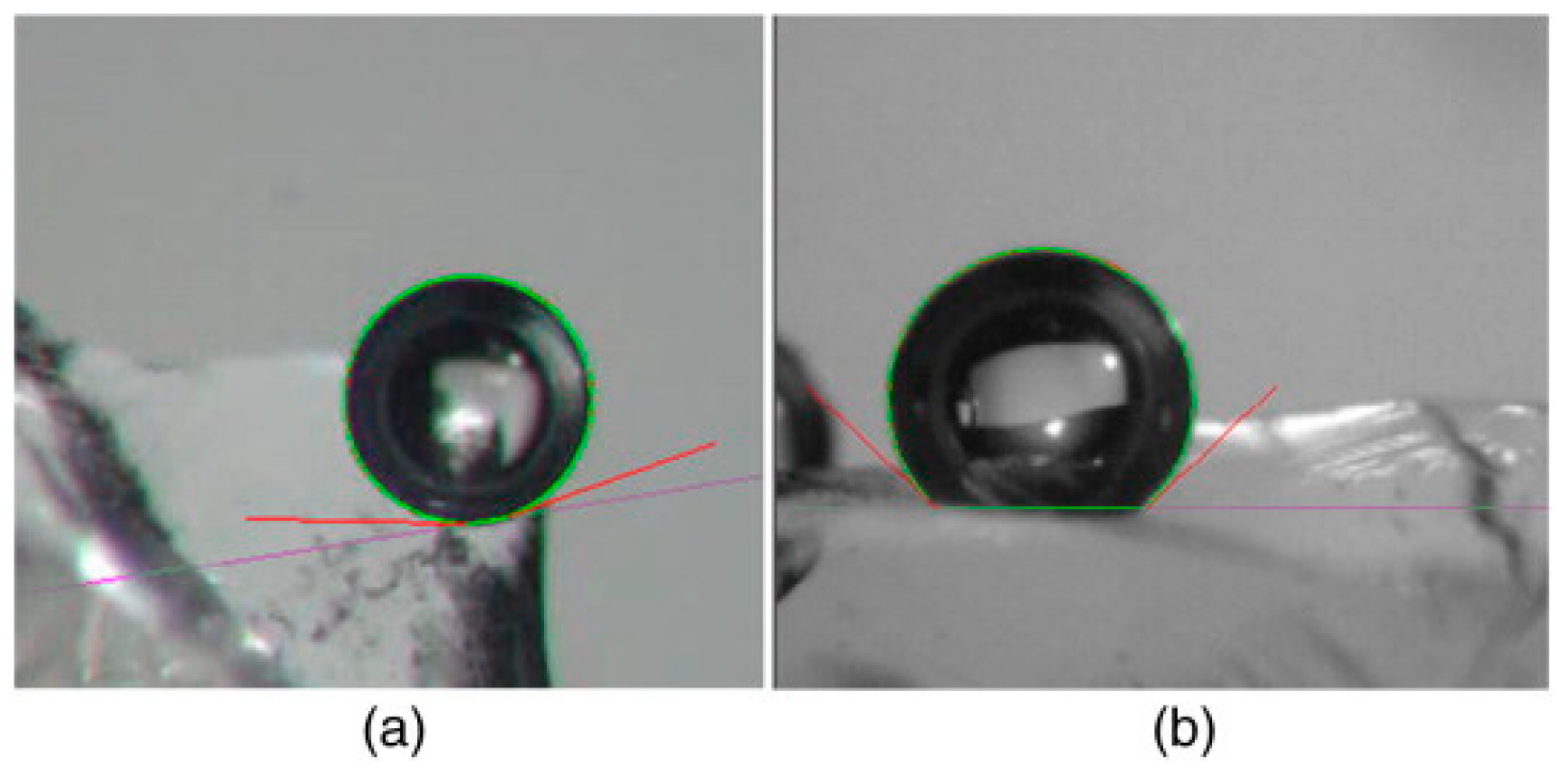
The difference in macroscopic bubble contact angle on quartz surface with or without nanobubbles [27].

It is worth adding that the nanobubbles can amplify the contact Angle of the surface of the hydrophobic particles. Ishida et al. reported that the apparent contact Angle of nanobubbles (160°) was much larger than that of macrobubble (70°) [25]. Song et al. observed the nanobubbles on the surface of hydrophobic binary SAMs were obviously very flat and expanded horizontally [26]. The contact Angle of the nanobubbles (145.2°) was significantly higher than that of the macrobubbles (60°), which is consistent with the study of Ishida et al. In addition, some researchers have indirectly demonstrated that nanobubbles can indeed change the surface hydrophobicity of hydrophobic particles and, thus, improve flotation selectivity. Calgaroto et al. showed that the contact angle of macrobubbles increased from 18° to 46° due to the existence of nanobubbles on the surface of quartz treated with amine (Figure 7) [27] that is, surface nanobubbles could enhance the hydrophobicity of mineral surfaces and improve the selectivity of flotation.

### 3.3. Pulp Particle Size

**Figure 8:**
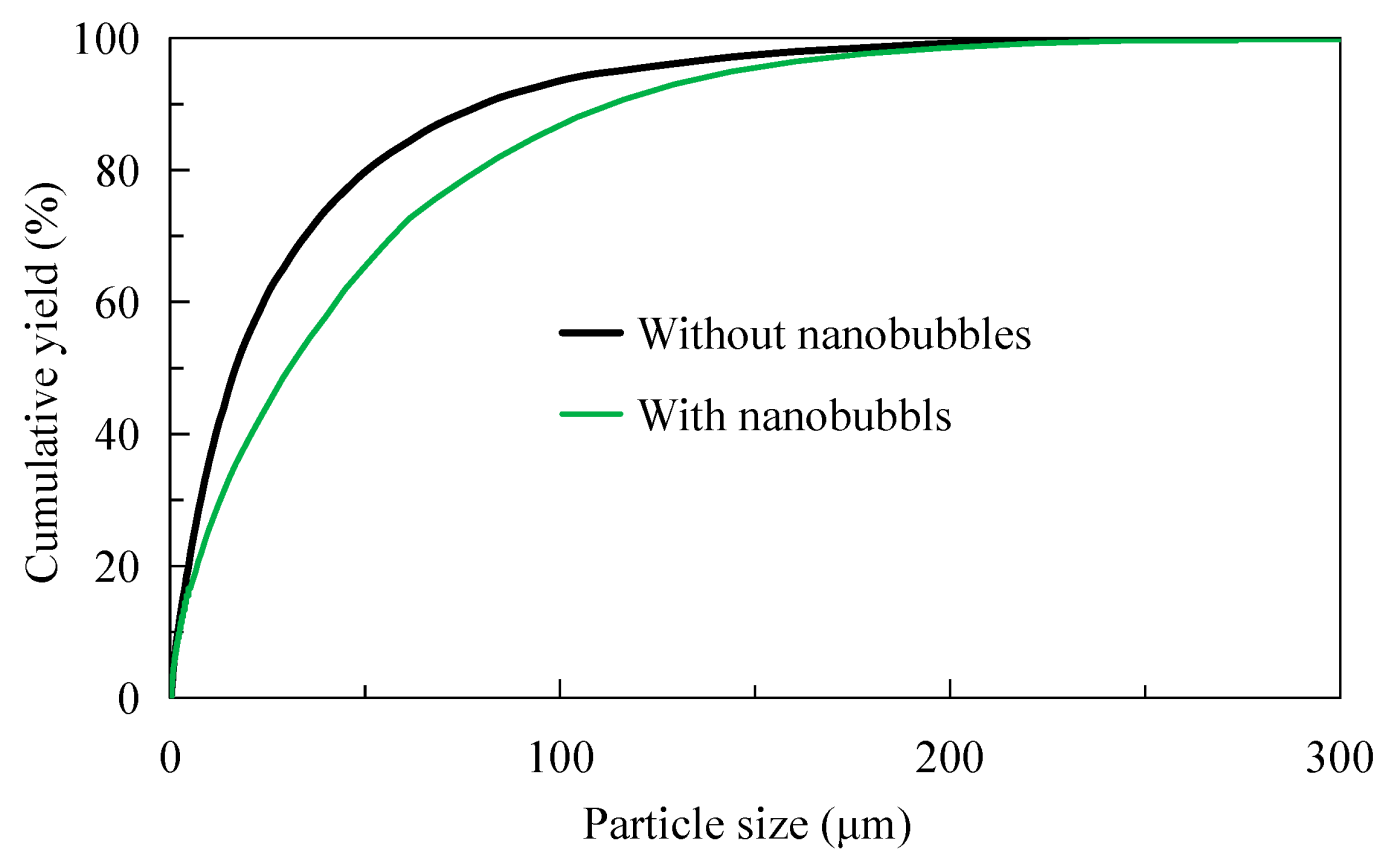
Particle size difference in graphite pulp with or without nanobubbles.

Figure 8 shows the difference in particle size between traditional flotation pulp and nanobubble flotation pulp. It can be seen from Figure 8 that the particle size of nanobubble flotation pulp is always larger than that of traditional flotation pulp. For example, when the size range is less than 100 μm, the cumulative particle size yield of nanobubble flotation pulp is about 86%, and that of traditional flotation pulp is about 93%; that is, the cumulative particle size yield of nanobubble flotation pulp is 7 percentage points lower than that of traditional flotation pulp. For the average size d50, the d50 in the nanobubble flotation pulp is 30 μm and the d50 in the traditional flotation pulp is 17 μm. The average size of the nanobubble flotation pulp is 13 μm higher than that in the traditional flotation pulp, which indicates nanobubble has an obvious agglomeration effect on the particles in the pulp. At present, this agglomeration phenomenon is caused by the capillary force between nanobubbles on the particle surface, which increases the apparent size of fine particles and thereby enhances the capture probability of bubbles and fine particle minerals.

**Figure 9:**
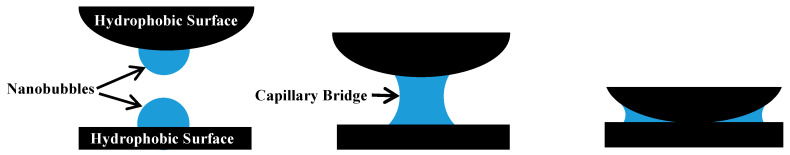
Formation process of bridging effect of nanobubble between hydrophobic surfaces [28].

Nanobubbles have an obvious hydrophobic agglomeration effect on the fine particles; previous studies have shown the two nanobubbles on the hydrophobic surface are the main factor causing the capillary force on the two hydrophobic surfaces. Hampton and Nguyen found that the nanobubbles would form a “bridge” between the two surfaces when the distance between two hydrophobic surfaces reached a certain level, which would promote the two surfaces to be attracted together by capillary force (Figure 9) [28]. In fact, there is no study that can completely confirm that nanobubbles are the only reason for the capillary force between two hydrophobic interfaces. However, the attraction between two hydrophobic interfaces with nanobubbles is stronger than that without nanobubbles. Therefore, nanobubbles are currently considered to be the reason for the capillary force between two hydrophobic interfaces at present [9]. However, the nature of the two capillary forces between the two hydrophobic interfaces is not clear. Ma et al. suggested that the capillary forces are a combination of DLVO forces and non-DLVO forces [21].

**Figure 10:**
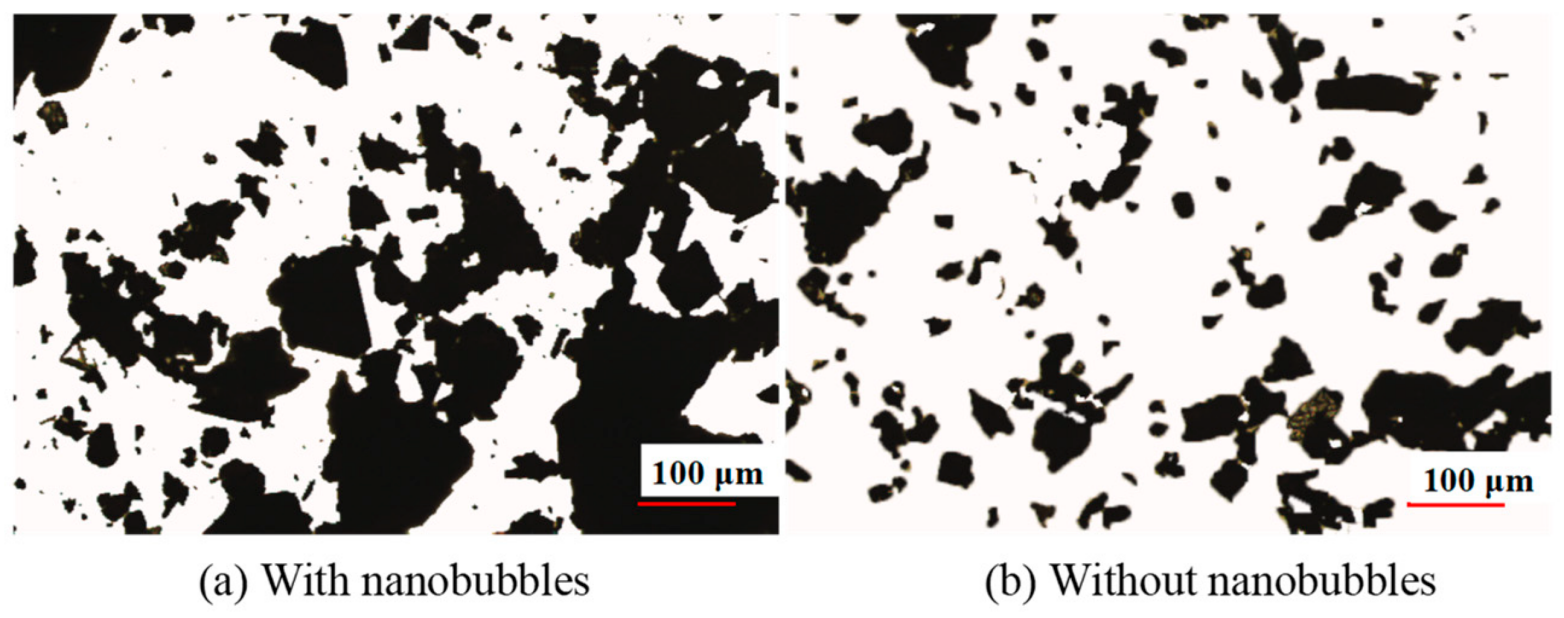
Morphological difference in fine flake graphite in pulp with or without nanobubbles [22].

In flotation, the nanobubbles on the hydrophobic particle surface can promote the fine particles to be firmly agglomerated together by this capillary force to form larger hydrophobic aggregates. For example, Tang et al. found through optical microscopy that the agglomeration effect of graphite flotation pulp was more obvious than that of traditional flotation (Figure 10) [23].

The stability of hydrophobic aggregates of fine particles is very important for flotation efficiency. In the range of floating particle size, the recovery effect of fine graphite can be improved by maintaining large apparent-size aggregates of micro-fine graphite. On the contrary, the difficulty of mineralization for micro-fine graphite recovery will be greatly increased if the structure of hydrophobic aggregates is not stable. At present, the strong stability of such hydrophobic aggregates has been proved by many researchers. For example, Dr. Ma’s study showed that the hydrophobic aggregates formed by micro-fine flake graphite promoted by nanobubbles are difficult to destroy even under ultrasonic environments [2]. At present, the reasons for the stability of hydrophobic aggregates can be summarized into two aspects. On the one hand, the nanobubbles on the graphite surface can reduce the electrostatic repulsion between two graphite particles, which is conducive to the stability of graphite hydrophobic aggregates [21]. On the other hand, the nanobubbles improve the surface hydrophobicity of graphite and enhance the hydrophobic attraction among graphite particles, thus promoting the stability of hydrophobic aggregates [22]. Furthermore, the stability of hydrophobic aggregates is the result of electrostatic force and hydrophobic attraction. According to the description of the flotation probability equation, when the flotation particle size is within a certain range, the hydrophobic agglomeration with larger apparent size can promote the enhancement of flotation collision probability and adhesion probability [17]. Therefore, the stable structure of the hydrophobic aggregates of fine graphite can improve the efficient recovery of micro-fine flake graphite.

### 3.4. Concentrate Particle Size

**Figure 11:**
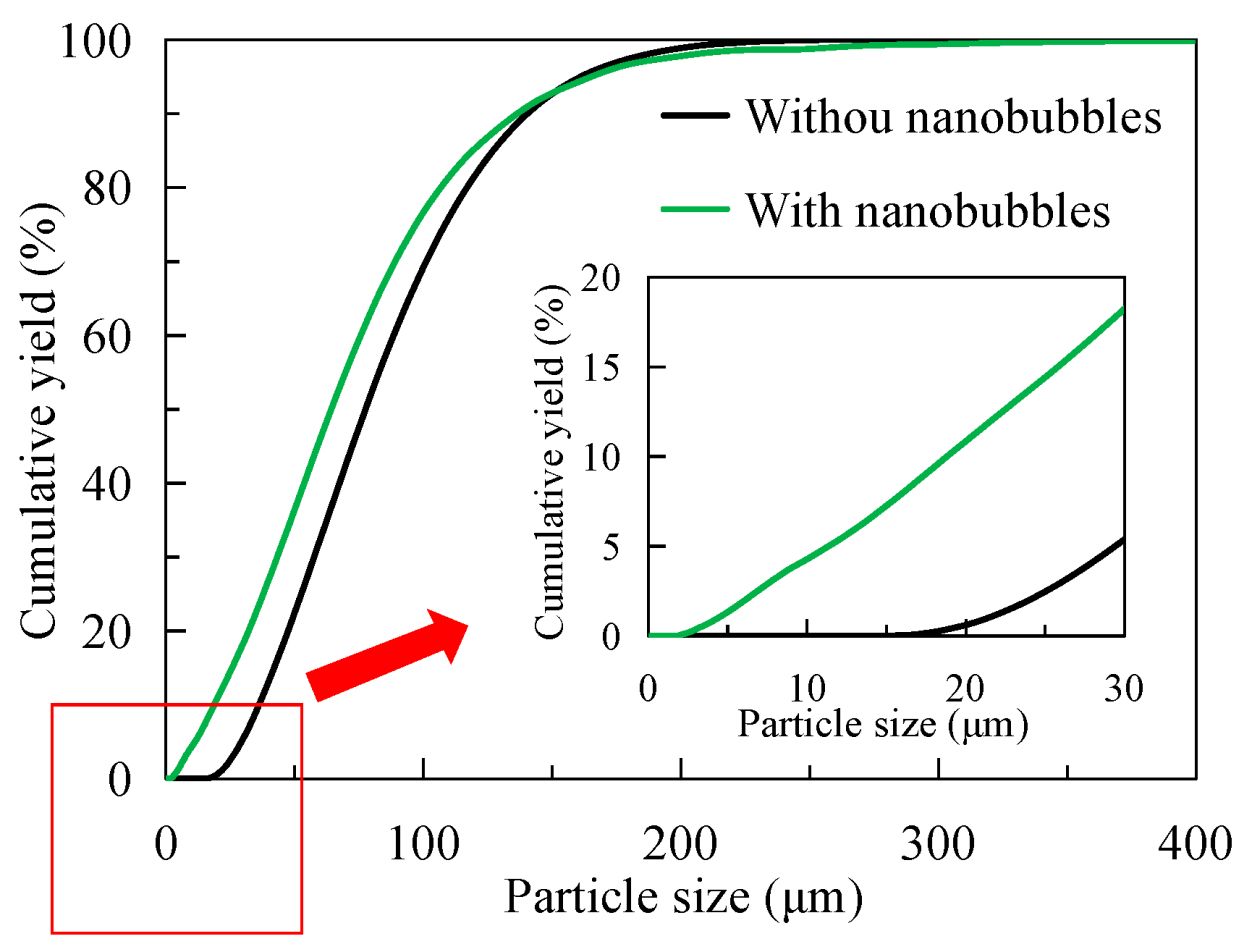
Particle size difference in graphite concentrate with or without nanobubbles.

Figure 11 shows the difference in particle size analysis between traditional flotation concentrate and nanobubble flotation concentrate after drying. It can be seen from Figure 11 that the particle size of nanobubble flotation concentrate is obviously finer than that of traditional flotation concentrate. The average particle size of nanobubble flotation concentrate is d50 = 65 μm, while that of traditional flotation concentrate is d50 = 78 μm; that is, the average particle size of nanobubble flotation concentrate is 13 μm smaller than that of traditional flotation concentrate. In addition, it can be seen from Figure 11 that the minimum particle size of the nanobubble flotation concentrate is about 2 μm, while that of the traditional flotation concentrate is about 16 μm. In other words, the existence of nanobubbles reduces the lower limit of flotation particle size and can effectively promote the recovery of microfine and even ultrafine (−10 μm) particle minerals. The particle size of microfine graphite particles recovered by nanobubble flotation is smaller, and the same conclusion was confirmed by Wang et al. in the flotation of ultrafine molybdenite [29]. Combined with the content in Section 3.3, nanobubbles can promote the agglomeration of fine particles in the pulp in the form of larger apparent size then these fine particles enter the foam layer as larger apparent size aggregates to become concentrates. Finally, the hydrophobic agglomeration effect of the concentrate disappears after drying, and the fine particles exist as independent individuals, so the particle size of the concentrate is finer than that of the traditional flotation concentrate. It is the remarkable enhanced recovery effect of nanobubbles on micro-fine flake graphite that makes the concentrate recovery higher, which is completely consistent with the test results shown in Figure 4 in Section 3.1.

### 3.5. Zeta Potential

**Figure 12:**
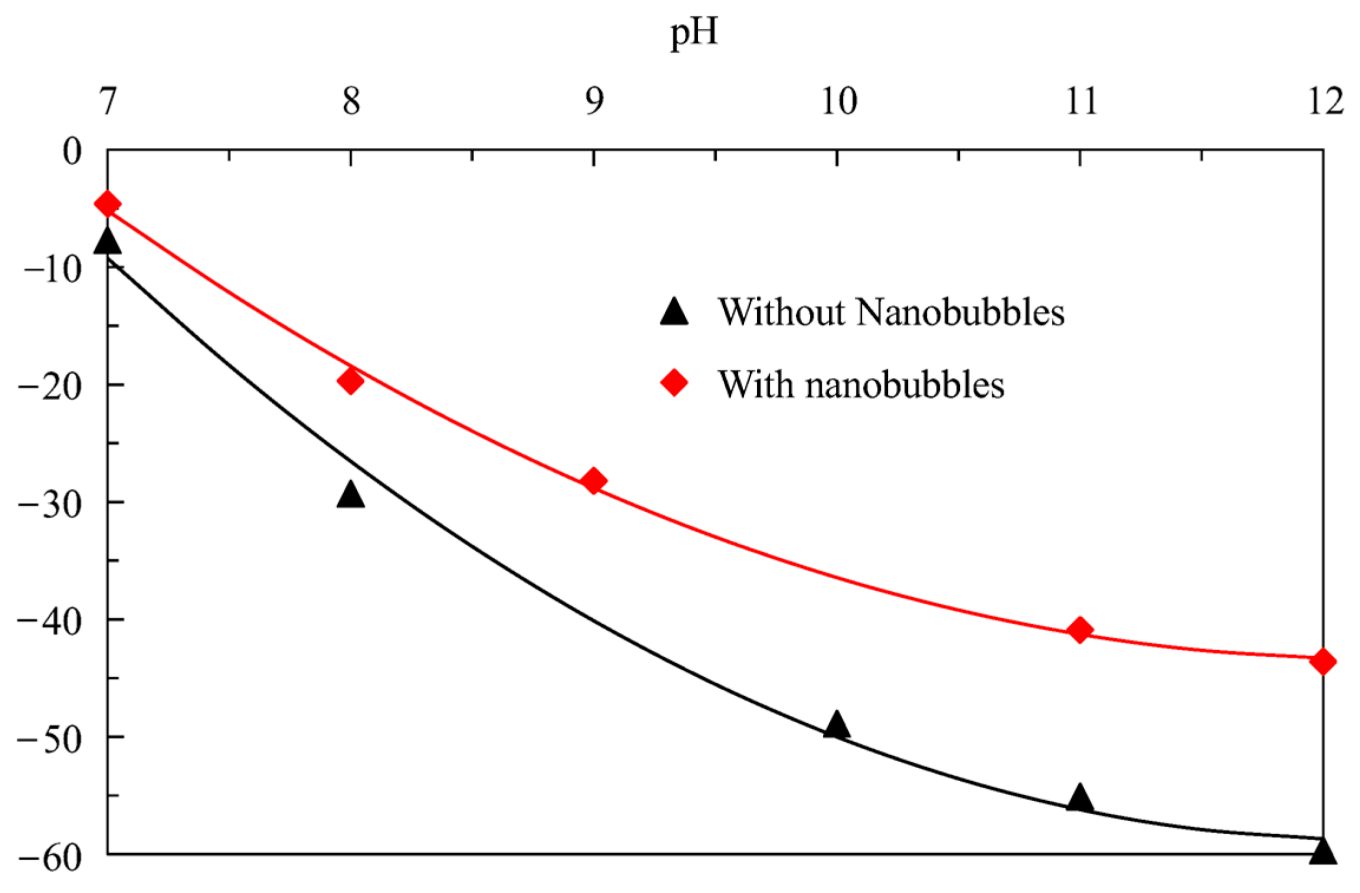
The surface potential difference in graphite in the presence or absence of nanobubbles.

Figure 12 shows that the difference in the surface potential of graphite is very obvious in the absence and presence of nanobubbles; that is, the surface potential of graphite is significantly reduced when nanobubbles are present. For example, the surface potential of graphite in the presence of nanobubbles is approximately −40.9 mV when the pH is around 11, while the surface potential of graphite reaches −55.1 mV in the absence of nanobubbles; that is, the presence of nanobubbles reduces the surface potential of graphite by 14.2 mV. With the increase in pH, the potential difference on the graphite surface becomes larger and larger. From the perspective of electrostatic adsorption, the negative potential of the graphite surface is relatively large in the absence of nanobubbles, showing a strong negative charge; that is, there is a significant electrostatic repulsive force between diesel and the graphite surface. On the contrary, the adsorption of nanobubbles on the graphite surface reduces the negative potential on the surface of graphite, indirectly weakening the electrostatic repulsion between graphite particles. In other words, the nanobubbles on the surface of graphite reduce the electrostatic repulsive force between graphite particles. It is well known that nanobubbles can promote the agglomeration of fine hydrophobic particles, increase the apparent size of fine particle graphite, and enhance the flotation probability of fine particle graphite by bubbles. The reduction in electrostatic repulsion between graphite particles is conducive to maintaining a stable structure of fine particle graphite aggregates, thereby ensuring that fine graphite maintains a large apparent size and providing favorable conditions for improving the conventional bubbles to facilitate graphite recovery. In fact, it has also been found that nanobubbles can effectively reduce the potential on the surface of minerals in the research of other minerals, such as pyrite [30], rutile [31], and argentite [32].

### 3.6. XPS

**Figure 13:**
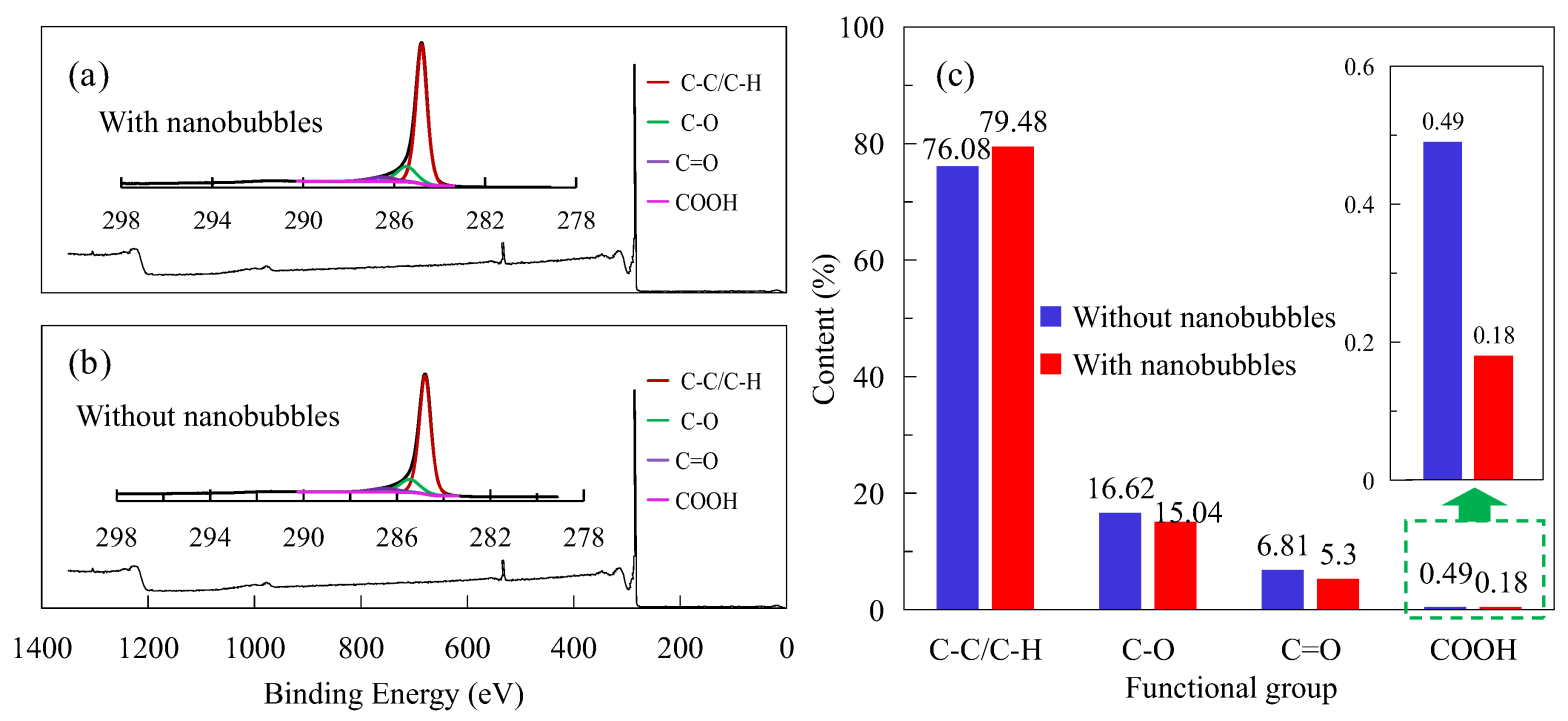
The results of XPS analysis in absence and presence of nanobubbles. (**a**) The specific corrections are as followsof the C peak when nanobubbles are present. (**b**) The results of the peak splitting of the C peak when nanobubbles do not exist. (**c**) The differences in hydrophilic and hydrophobic groups under the presence or absence of nanobubbles.

Figure 13a,b show the XPS analysis results of the flotation pulp with and without nanobubbles, respectively, presenting the wide peak of XPS and splitting peak results of peak C. The contents of hydrophilic groups (such as C-O, C-O, C=O, and COOH) and hydrophobic groups (such as C-C/C-H) with and without nanobubbles were obtained (see Figure 13c) based on the peak separation results. It can be seen from Figure 13c that the C-C/C-H content without nanobubbles is 77.28%, and the C-C/C-H content with nanobubble is 79.48%; that is, the C-C/C-H content on the graphite surface with the nanobubbles sample has increased by 3.40%. In addition, compared with in the absence of nanobubbles, the contents of the C-O, C=O, and COOH on the graphite surface with nanobubbles decreased by 1.58%, 1.51%, and 0.31%, respectively. C-C/C-H are hydrophobic functional groups, while C-O, C=O, and COOH are hydrophilic functional groups. In other words, the content of hydrophobic functional groups on the graphite surface significantly increased in the presence of nanobubbles, while the content of hydrophilic functional groups significantly decreased.

The increase in the content of hydrophobic functional groups on the graphite surface is derived from C-C/C-H in diesel molecules, which indicates that nanobubbles effectively promote the adsorption of diesel on the graphite surface. The existence of nanobubbles effectively covers the hydrophilic functional groups on the surface of graphite, resulting in a decrease in the content of oxygen-containing functional groups on the surface of graphite. The reduction in hydrophilic group content can provide more active sites for the collector and secondarily enhance the hydrophobicity of the graphite surface to improve the hydrophobicity difference between graphite and gangue. In fact, Wang et al. also found in their mineral research that nanobubbles can form preferentially on the surface of galena rather than pyrite, amplifying the hydrophobic difference between the surfaces of galena and pyrite and improving the separation efficiency [33]. Wu et al. [30] also found in the study of nanobubbles on the pyrite surface that the coverage of nanobubbles on the pyrite surface can reduce the oxidation rate of the pyrite surface, improve the adsorption capacity of sodium amyl xanthate on the surface of pyrite, and ensure that the surface of pyrite maintains a high hydrophobicity. The above research results are completely consistent with this study. It should be noted that enhancing the hydrophobicity of the graphite surface can increase the hydrophobic attraction between graphite particles, making the hydrophobic aggregates caused by nanobubbles more stable. This ensures that fine particles are captured by the bubbles, thereby achieving the effect of strengthening the recovery of fine-particle graphite.

## 4. Conclusions

(1) The nanobubble flotation rate is significantly higher than that of conventional flotation, especially within 15 s of flotation. In addition, the recovery of nanobubble flotation concentrate is significantly higher than that of traditional flotation concentrate, and the final concentrate recovery is about 5 percentage points higher than that of traditional flotation concentrate.
(2) Nanobubble flotation selectivity is better than traditional flotation. Under the same flotation time, the grade of flotation concentrate is always higher than that of traditional flotation concentrate. In addition, nanobubble flotation concentrate has a higher grade than traditional flotation concentrate with the same graphite recovery.
(3) Nanobubbles promote the agglomeration of fine graphite particles in the pulp, and the average particle size is 13 μm larger than that of the traditional flotation pulp. On the contrary, the particle size of nanobubble flotation concentrate is obviously finer than that of traditional flotation concentrate, and its average particle size is 13 μm smaller than that of traditional flotation concentrate. In addition, the minimum graphite size of nanobubble flotation is only 2 μm, 11 μm smaller than the minimum size of traditional flotation.
(4) Nanobubbles can reduce the electrostatic repulsion between fine particle graphite and enhance the hydrophobic attraction between graphite particles, which is conducive to maintaining the hydrophobic agglomeration structure of fine particle graphite and thereby strengthening the recovery of fine particle graphite. More importantly, nanobubbles can effectively cover the oxygen-containing groups on the surface of graphite, promoting the adsorption of collectors on the graphite surface and thereby enhancing the selectivity of fine particle graphite flotation.

## Figures and Tables

**Table 1:** Mineral composition in the sample.

Mineral	Graphite	Quartz	Pyrite	Calcite	Feldspar	Muscovite
Content (%)	28.09	26.01	6.40	7.32	5.30	16.32

## Data Availability

The original contributions presented in this study are included in the article. Further inquiries can be directed to the corresponding author(s).
